# Mandibular Brown Tumor as a Result of Secondary Hyperparathyroidism—Radiological and Clinical Pitfalls and Dilemmas

**DOI:** 10.3390/diagnostics15212798

**Published:** 2025-11-05

**Authors:** Ömer Uranbey, Furkan Diri, Büşra Ekinci, Michał Gontarz, Piotr Kuropka, Maciej Dobrzyński, Kamil Nelke

**Affiliations:** 1 Department of Oral and Maxillofacial Surgery, Faculty of Dentistry, Aydın Adnan Menderes University, Aydın 09100, Türkiye; furkandirifd@gmail.com; 2 Department of Medical Pathology, Faculty of Medicine, Aydın Adnan Menderes University, Aydın 09100, Türkiye; busra.ekinci@adu.edu.tr; 3 Department of Cranio-Maxillofacial Surgery, Jagiellonian University Medical College, 30-688 Cracow, Poland; 4 Division of Histology and Embryology, Department of Biostructure and Animal Physiology, Wrocław University of Environmental and Life Sciences, Cypriana K. Norwida 25, 50-375 Wrocław, Poland; 5 Department of Pediatric Dentistry and Preclinical Dentistry, Wrocław Medical University, Krakowska 26, 50-425 Wrocław, Poland; maciej.dobrzynski@umw.edu.pl; 6 Health Department, Angelus Silesius Academy of Applied Sciences in Wałbrzych, Zamkowa 4, 58-300 Wałbrzych, Poland

**Keywords:** bone, hyperparathyroidism, mandibular lesion, differential diagnosis, CBCT

## Abstract

Brown tumors (BTs) are rare osteolytic lesions that typically occur in association with primary or secondary hyperparathyroidism (PHP and SHP). Excessive secretion of parathyroid hormone induces increased bone resorption, resulting in lesions characterized by fibrosis, vascularization, and hemosiderin deposition. The most common sites include the jaws, ribs, pelvis, and long bones. Clinical manifestations may involve pain, swelling, or pathological fractures. We present the case of a mandibular BT in a 48-year-old female with chronic renal failure and secondary hyperparathyroidism. The patient exhibited progressive mandibular swelling with radiological features resembling an aggressive odontogenic or malignant lesion. Laboratory analysis confirmed markedly elevated parathyroid hormone levels, while scintigraphy demonstrated increased focal uptake in the mandible and ribs. Histopathological evaluation revealed multinucleated giant cells within a fibrous stroma, consistent with BT. Despite initiation of systemic endocrine therapy, the lesion continued to enlarge, necessitating complete surgical excision of the mandibular mass. This case underscores the diagnostic dilemmas of mandibular BT, which may closely mimic aggressive jaw pathologies. Importantly, while many BTs regress after systemic management of hyperparathyroidism, this case illustrates that surgical excision may be unavoidable in patients with unstable systemic status or progressive local disease. Comprehensive clinical, radiological, laboratory, and histopathological evaluation remains essential to ensure timely diagnosis and appropriate treatment.

**Figure 1:**
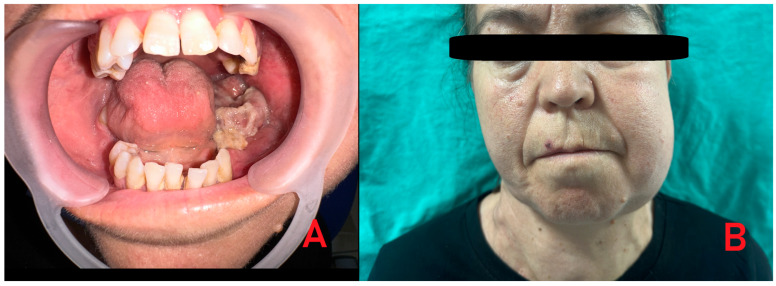
A 48-year-old female patient with a history of chronic renal failure on dialysis and regular use of anticoagulant and antihypertensive medications was referred for evaluation due to atypical mandibular pain, facial asymmetry, and swelling. Clinical examination revealed an irregular lesion in the left mandibular retromolar region with both intraosseous and exophytic components, accompanied by a foul odor suggestive of secondary infection. Typically, most cases of primary hyperparathyroidism (PHP) can be diagnosed relatively easily because of co-existing systemic morbidities, including bone symptoms (osteoporosis), urological signs (urolithiasis, polyuria, nocturia, hypercalciuria), gastrointestinal manifestations (loss of appetite, peptic ulcer), cardiovascular symptoms (hypertension, arrhythmia), neurological findings (muscle weakness), and psychological disturbances (headaches, depression, mood swings, sleep disorders). These features are mostly related to parathyroid over-secretion and subsequent calcium–phosphate–PTH imbalance. Since the patient was treated for a long time because of various general illnesses, with slowly progressive bone changes, the incidence of secondary hyperparathyroidism was suspected. Medical history revealed uncontrolled hypertension, chronic renal failure, and the patient undergoing dialysis three times per week. Heparin was administered on dialysis days, while aspirin was used on non-dialysis days. The patient also reported halitosis, which was confirmed during the clinical examination. Before surgery, the patient presented with swelling in the left facial region (**A**,**B**). Intraoral evaluation demonstrated that the pathological tissue was markedly fragile and prone to bleeding.

**Figure 2:**
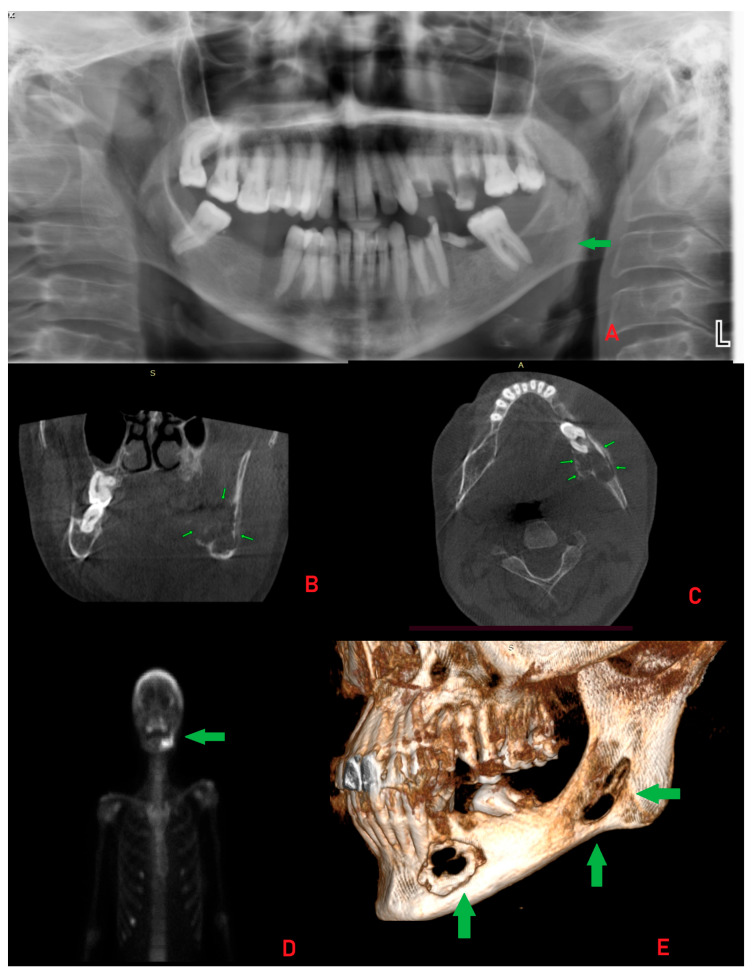
The panoramic radiograph revealed osteolytic areas, some of which were multilocular, in the left mandibular region without clear borders or shape ((**A**), L-left side). The residual roots of teeth 37 and 36 displayed a “floating teeth” appearance. A large radiolucent area extending toward the mandibular angle was observed, along with unilocular lesions extending anteriorly to the midline. The lesions resulted in thinning and erosion of the cortical bone. This radiographic appearance closely resembled that of ameloblastoma, central giant cell granuloma (CGCG), primary intraosseous carcinoma (PIOSC), plasmacytoma, or other malignant and non-malignant bone pathologies, including the rare possibility of malignant oncocytoma. Atypical in this case was the radiological and clinical presentation closely mimicking aggressive odontogenic or malignant lesions, despite the final diagnosis of a Brown Tumor (BT) associated with SHP [1,2,3]. Cone-beam computed tomography (CBCT) images demonstrated cortical expansion with marked thinning and bone loss (thin green arrows). Although no pathological fracture was identified (**B**,**C**), the patient reported discomfort while chewing and eating. Internal septa were evident, producing radiological patterns resembling “honeycomb” or “soap bubble” structures along with some calcified bodies in the lesion. The CBCT revealed an intraosseous lesion, measuring approximately 17 × 14 × 18 mm, that extended from teeth 33–36 toward the ramus, with irregular borders, buccal and lingual cortical perforation, irregular bone loss, and involvement of the mandibular canal. Despite this involvement, no lower lip numbness (Vincent’s sign) was present. The lesions exhibited irregular borders, and differential diagnoses also included traumatic bone cyst, Langerhans cell histiocytosis, plasmacytoma, and brown tumor (BT), primarily because of the extent of bone loss, irregular borders, bone structure changes, and a mixed radiolucent/radiopaque atypical structure on CBCT [2,4,5]. Improved diagnostics included whole-body scintigraphy and SPECT images after intravenous administration of 20 mCi Tc-99m MDP. The images showed diffuse increased uptake in the calvarium, the left hemimandible, and several ribs, most prominently at the anterior end of the right seventh rib (**D**). On the other hand, the scope of 3D-CBCT mandible reconstruction identified significant mandible cortical bone loss and an appearance like “moth-eaten” ((**E**), thick green arrows). The lesion’s radiological and clinical appearance was highly suggestive of aggressive odontogenic or malignant conditions, such as primary intraosseous squamous cell carcinoma (PIOSCC), osteosarcoma, plasmacytoma, or metastatic disease. Because of this atypical presentation, an incisional biopsy was prioritized before systemic markers were assessed, to exclude malignancy and establish a definitive histopathological diagnosis [4,5,6]. An incisional biopsy was performed under local anesthesia, and histopathological analysis revealed the presence of giant cells. Blood tests were obtained, which supported but did not definitively confirm the diagnosis (Supplementary Materials). At baseline, prior to the incisional biopsy, serum PTH (1070.6 pg/mL) and ALP (381 U/L) levels were markedly elevated, while calcium (9.3 mg/dL) and phosphorus (2.6 mg/dL) remained within or slightly below the reference range, indicating an early biochemical profile consistent with hyperparathyroidism. The patient was subsequently referred to the endocrinology and nephrology departments for further evaluation, including parathyroid function testing. Considering these findings and the patient’s systemic condition, SHP was confirmed. Medical treatment was initiated, leading to limited regression of the lesion and swelling, though complete remission was not achieved.

**Figure 3:**
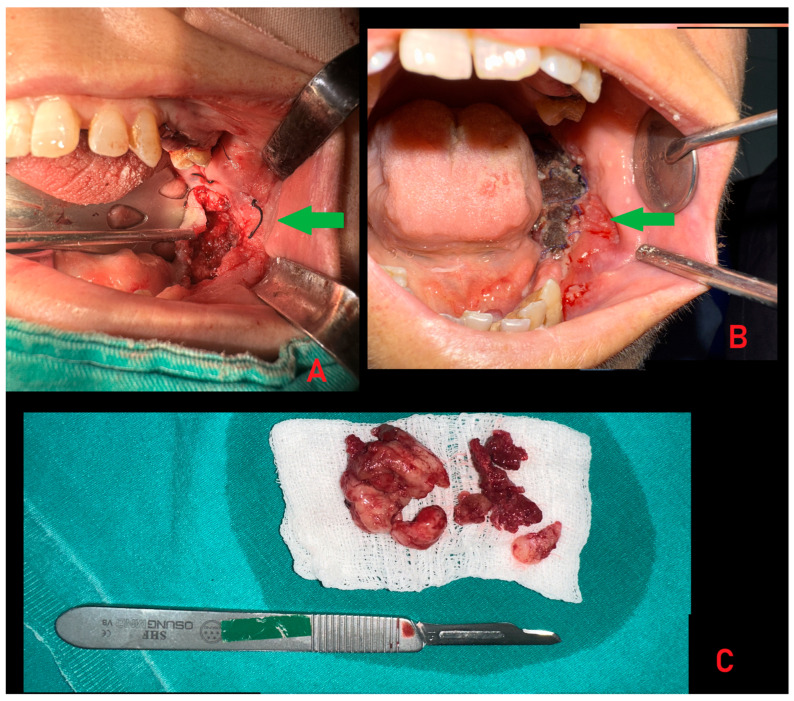
In most patients, general management of secondary hyperparathyroidism, including parathyroidectomy with subsequent endocrine regulation, leads to stabilization or regression of BT. In the present case, however, neither medical treatment nor systemic regulation achieved sufficient control of the lesion. Therefore, a decision was made to perform a complete surgical excision of the mandibular mass, accompanied by additional biopsies to exclude any coexisting pathology or alternative diagnosis. Subsequently, the patient was scheduled for general surgery consultation and possible parathyroidectomy. Despite the initiation of treatment by endocrinology and nephrology, only minimal regression of the patient’s lesion was observed. Therefore, an excisional biopsy under general anesthesia was planned. Given the patient’s elevated risk of bleeding, heparin therapy was discontinued during the dialysis session preceding the surgical intervention. Intraoperatively, vessels exhibiting active bleeding were circumferentially ligated with sutures. The lesion was excised in its entirety down to sound bone ((**A**,**B**), green arrows). Bleeding foci were managed with cauterization, and teeth associated with the lesion margins were extracted. Sponges were applied as a tamponade to achieve hemostasis. Curettage of the surrounding bone was performed until intact bone walls were reached (**C**). Finally, the lesion cavity was covered with oxidized regenerated cellulose (Surgicel, Ethicon, Johnson & Johnson, Los Frailes, Bronx, NY, USA) and closed with sutures to ensure hemostatic control. The substantial thickness of the remaining bone walls confirmed the absence of fracture risk. Some authors advise performing only a biopsy, whereas others recommend an excisional biopsy to obtain a more representative sample for histopathological evaluation. In contrast, some clinicians prefer to wait for spontaneous bone healing or to use buccal fat pads to cover any oro-nasal or oro-antral communications [6].

**Figure 4:**
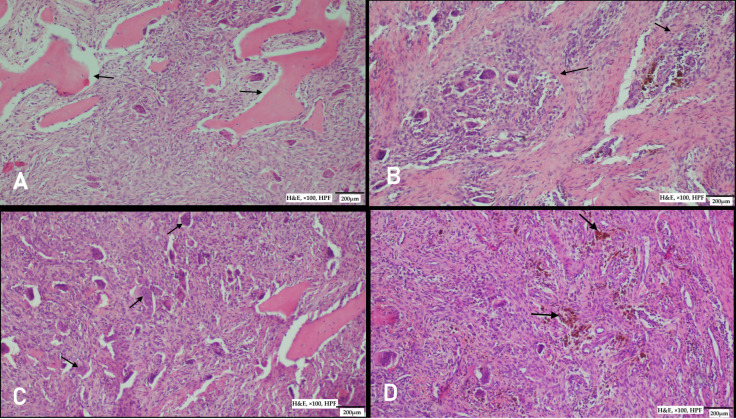
Histopathological examination revealed characteristic bone alterations. (**A**) Tunnel-shaped resorption areas were observed surrounding adjacent, non-affected bone tissue, reflecting the osteoclastic activity within the lesion (tunnel resorption areas are marked with arrows). (**B**) The lesion was predominantly composed of osteoclast-type giant cells arranged in indistinct nodular patterns (nodular areas marked with arrows). (**C**,**D**) Within the background, a vascular fibroblastic stroma was noted, containing multiple groups and clusters of osteoclast-type giant cells. These findings are consistent with giant cell–rich lesions typically associated with hyperparathyroidism or reparative granulomatous processes. (**C**) Arrows indicate osteoclast-type giant cells; (**D**) arrows indicate hemosiderin deposits (H&E, ×100, HPF).

**Figure 5:**
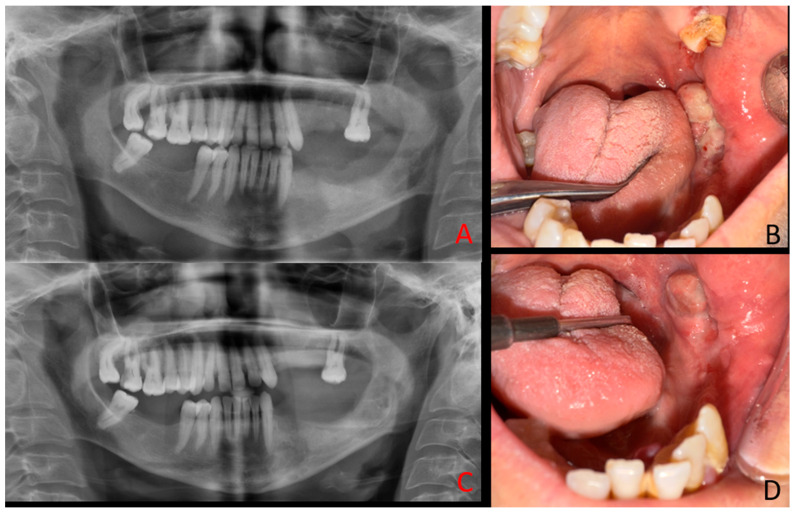
BTs are well-recognized skeletal manifestations of hyperparathyroidism, yet their occurrence in the mandible remains rare, particularly in the context of secondary hyperparathyroidism [7,8]. The present case is also distinctive for its unusual clinical course: unlike most BTs that regress following systemic management [9], the lesion demonstrated relentless progression and ultimately required complete surgical excision. Moreover, the clinical and radiological presentation closely mimicked aggressive odontogenic or malignant pathology. Clinically, the patient exhibited an exophytic, ulcerated mandibular mass with irregular borders and secondary infection, while radiologically, the multilocular osteolytic pattern with cortical perforation resembled ameloblastoma, central giant cell granuloma, primary intraosseous carcinoma, or osteosarcoma. This convergence of rarity, malignant-like presentation, and resistance to conventional endocrine therapy underscores the diagnostic and therapeutic dilemmas posed by mandibular BTs in secondary hyperparathyroidism. Sequential intraoral photographs and panoramic radiographs were obtained at 2 months and 4 months postoperatively. At 2 months, early signs of healing at the surgical site are evident (**A**,**B**). By 4 months, further bone remodeling and soft tissue recovery can be observed (**C**,**D**). The patient remains under regular medical and clinical follow-up to monitor changes in both intraoral and radiological appearance of the lesion. It should be noted that bone lesions accompanied by mucosal or gingival ulcerations, swelling, or erosions may mimic benign/malignant tumors rather than primary or secondary hyperparathyroidism, including central giant cell granuloma, ameloblastoma, and primary intraosseous squamous cell carcinoma. A mandibular lesion was initially diagnosed as a giant cell tumor before recognition as a Brown tumor [1], while another case presented with malignant-like features in the mandible [10]. Diagnostic confusion has also extended to lesions first interpreted as metastatic disease [11]. Radiological appearances such as multilocular “soap-bubble” or “moth-eaten” patterns, cortical perforation, and irregular borders are therefore not pathognomonic, underscoring the need for biochemical correlation with parathyroid hormone and alkaline phosphatase levels. Even with systemic control, persistence of aggressive jaw lesions has been reported after parathyroidectomy, necessitating surgical excision [12]. Further evidence confirms this overlap. Multiple destructive head-and-neck lesions were described in hyperparathyroidism, initially suggesting aggressive odontogenic disease [13]. Brown tumors of the mandible have repeatedly been mistaken for malignancies [10,14], while in other sites, lesions were treated as malignancies until endocrine testing revealed the true diagnosis [15]. More recently, histopathological resemblance to central giant cell granuloma has been documented in mandibular cases [16].

**Figure 6:**
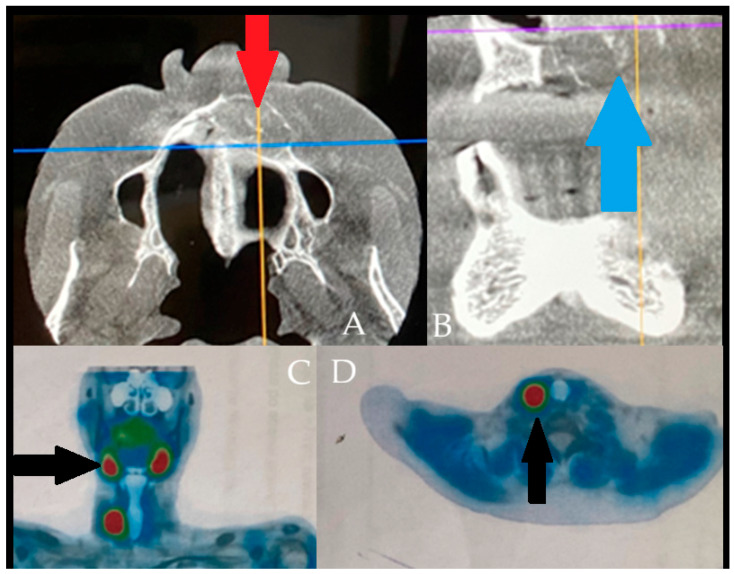
The following figure is a comparable case image to the presented case; however, the BT lesions found were not that advanced locally and were misdiagnosed as other minor jaw lesions. However, the performance of SPECT-MiBi improved greatly on the final diagnosis. The occurrence of BT is a very troublesome disease in identification in some atypical cases, especially in small lesions, without significant aggressive bone loss, extracortical spread, and when they have a similar appearance to a normal jaw cyst [8,9]. The differential diagnostics might be difficult, when some bone lesions in jaw bones can mimic a vast scope of other tumors and lesions of both benign and malignant origins ((**A**,**B**) with blue-orange and purple-orange orientation lines). Sometimes, regardless of a full blood-work and evaluation of calcium-phosphatase markers, very good radiology, the full scope of the disease might often be camouflaged by other general patient co-morbidities. In cases of any doubts, a special scintigraphy of the parathyroid glands (SPECT-MiBi) (Black arrows, (**C**,**D**)) is very important, along with a biopsy to fully confirm this disease [2,4,8]. The scope of BT in PHP/SHP might have many levels of bone changes, blood serum markers exhibition, and, therefore, histopathology. Acquiring more valuable material for accurate histopathology is quite important. The following factors may be associated with various bone changes, the spectrum of lesions, and osteolytic processes detected within a single biopsy specimen. BT can manifest in various ways and with different possible radiological appearances, which are greatly dependent on the scope, intensity, and duration of PHP/SHP [4,5,6,7]. The scope of the surgical approach towards the parathyroid glands and thyroids, if necessary, should be discussed among endocrinologists and general surgeons after a careful individual examination of each case. Collectively, these reports highlight that BT of the jaws is diagnostically challenging due to their overlap with odontogenic and malignant lesions. Accurate diagnosis requires correlation of imaging with biochemical and histopathological findings, and surgical excision may be necessary when systemic therapy proves insufficient.

## Data Availability

The data presented in this study are available on request from the corresponding author due to privacy.

## Supplementary Materials

The following supporting information can be downloaded at: https://www.mdpi.com/article/10.3390/diagnostics15212798/s1, Figure S1: Biochemical markers at three time points T1, T2 and T3; Table S1: Biochemical markers at three time points T1, T2 and T3.
