# Supplementary material for: Mandibular Brown Tumor as a Result of Secondary Hyperparathyroidism—Radiological and Clinical Pitfalls and Dilemmas
Source: Diagnostics (Basel). 2025 Nov 5;15(21):2798. doi: 10.3390/diagnostics15212798 (PMC12609294; doi:10.3390/diagnostics15212798)
Supplement: Supplementary file 1 [file diagnostics-15-02798-s001.zip › diagnostics-3953422-supplementary.pdf]

diagnostics

MDPI

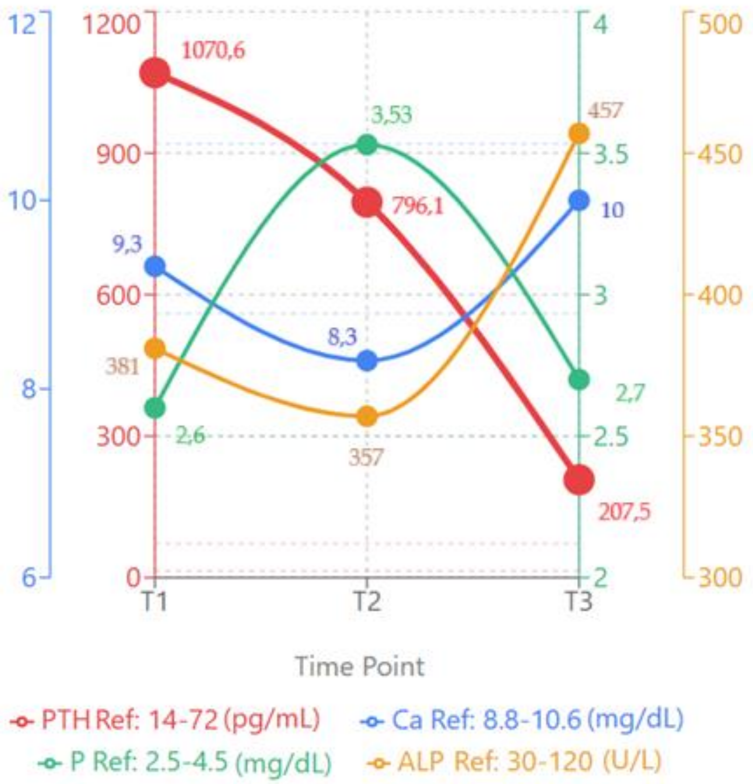

Figure S1. Biochemical markers at three time points T1, T2 and T3.

Figure Abbreviations: At T1 (preoperative, before incisional biopsy), PTH and ALP were markedly elevated. At T2 (preoperative, before establishing the diagnosis of brown tumor), PTH remained high with persistently increased ALP and mild changes in calcium and phosphorus. At T3 (postoperative), PTH showed a marked decline with normalization of calcium and phosphorus, while ALP remained elevated, indicating ongoing bone remodeling. PTH, parathyroid hormone; Ca, calcium; P, phosphorus; ALP, alkaline phosphatase.

Table S1. Biochemical markers at three time points T1, T2 and T3.

Markers	T1	T2	T3	Reference Value
**PTH (pg/mL)**	1070.6	796.1	207.5	14-72
**Ca (mg/dL)**	9.3	8.3	10	8.8-10.6
**P (mg/dL)**	2.6	3.53	2.7	2.5-4.5
**ALP (U/L)**	381	357	457	30-120

***Abbreviations: At T1 (preoperative, before incisional biopsy), PTH and ALP were markedly elevated. At T2 (preoperative, before establishing the diagnosis of brown tumor), PTH remained high with persistently increased ALP and mild changes in calcium and phosphorus. At T3 (postoperative), PTH showed a marked decline with normalization of calcium and phosphorus, while ALP remained elevated, indicating ongoing bone remodeling. PTH, parathyroid hormone; Ca, calcium; P, phosphorus; ALP, alkaline phosphatase.
